# Human neonatal rotavirus vaccine (RV3-BB) targets rotavirus from birth

**DOI:** 10.1056/NEJMoa1706804

**Published:** 2018-02-22

**Authors:** Julie E. Bines, Jarir At Thobari, Cahya Dewi Satria, Amanda Handley, Emma Watts, Daniel Cowley, Hera Nirwati, James Ackland, Jane Standish, Frances Justice, Gabrielle Byars, Katherine J. Lee, Graeme L. Barnes, Novilia S. Bachtiar, Ajeng Viska Icanervilia, Karen Boniface, Nada Bogdanovic-Sakran, Daniel Pavlic, Ruth F. Bishop, Carl D. Kirkwood, Jim P. Buttery, Yati Soenarto

**Affiliations:** 1 RV3 Rotavirus Vaccine Program, Murdoch Childrens Research Institute, Parkville, Victoria, Australia (J.E.B., A.H., D.C., E.W., C.D.K., J.S., K.J.L, F.J., G.B., K.B., N.B., D.P., G.L.B., R.F.B., J.P.B.); Department of Paediatrics, The University of Melbourne (J.E.B., D.C., C.D.K., K.J.L., G.L.B., R.F.B., J.P.B.); Department of Gastroenterology and Clinical Nutrition, Royal Children's Hospital (J.E.B., J.S.); 2 Department of Paediatrics, The University of Melbourne (J.E.B., D.C., C.D.K., K.J.L., G.L.B., R.F.B., J.P.B.); 3 Department of Gastroenterology and Clinical Nutrition, Royal Children's Hospital (J.E.B., J.S.); 4 Pediatric Research Office, Department of Paediatrics (C.D.S, A.V.I., Y.S.), Department of Pharmacology and Therapy (J.A.T.) and Department of Microbiology (H.N.W.); Faculty of Medicine, Universitas Gadjah Mada, Yogyakarta; 5 Medicines Development for Global Health, Melbourne, Australia (A.H); 6 Global BioSolutions, Melbourne, Australia (J.A.); 7 PT Bio Farma, Bandung, Indonesia (N.S.B.); 8 Bill and Melinda Gates Foundation, Seattle, U.S.A. (C.D.K.); 9 Departments of Paediatrics and Epidemiology & Preventive Medicine, Monash University; Department of Infection & Immunity, Monash Children’s Hospital, Clayton, Victoria, Australia (J.P.B.)

## Abstract

**Background:**

A birth dose strategy using a neonatal rotavirus vaccine to target early prevention of rotavirus disease may address remaining barriers to global vaccine implementation.

**Methods:**

We conducted a randomized, placebo-controlled trial in Indonesia to evaluate the efficacy of an oral human neonatal rotavirus vaccine (RV3-BB) to prevent rotavirus gastroenteritis. Healthy newborns received three doses of RV3-BB administered in a neonatal schedule at 0-5 days, 8 and 14 weeks or infant schedule at 8, 14 and 18 weeks, or placebo. Laboratory-confirmed rotavirus gastroenteritis was graded using a modified Vesikari score. The primary analysis was efficacy against severe rotavirus gastroenteritis from two weeks after all doses to 18 months in the combined vaccine group (neonatal and infant schedule) compared with placebo.

**Results:**

Vaccine efficacy against severe rotavirus gastroenteritis to 18 months was 63% in the combined vaccine group (95% CI 34, 80; p<0.001), 75% in the neonatal vaccine group (95% confidence interval [CI] 44, 91; p<0.001) and 51% in the infant vaccine group (95% CI 7, 76; p=0.03) in the per protocol analysis, with similar results in the intention-to-treat analysis. Vaccine efficacy to 12 months was 94% in the neonatal vaccine group (95%CI 56, 99; p=0.006). Vaccine take occurred in 78/83 (94%) in the neonatal vaccine group and 83/84 (99%) in the infant vaccine group. The vaccine was well tolerated, with similar incidence of adverse events in vaccine and placebo recipients.

**Conclusion:**

RV3-BB was efficacious, immunogenic and well-tolerated when administered in a neonatal or infant schedule in Indonesia.

## Background

Despite evidence of success of rotavirus vaccines, over 90 million infants still lack access to a rotavirus vaccine (1, 2). Barriers to global implementation include cost, sub-optimal efficacy in low-income countries and lingering safety concerns (3, 4). An oral rotavirus vaccine administered at birth has potential to address these challenges.

Rotavirus disease occurs early in life in infants in low-income countries (5). A birth dose rotavirus vaccine would provide early protection and maximize the opportunity to complete a full vaccine schedule (6). Birth presents a unique opportunity that may assist the uptake of an oral vaccine as gastric acid is limited and environmental enteropathy not yet established (7, 8). As intussusception is rare in newborns, a birth dose administration may offer a safety advantage (9).

RV3-BB vaccine was developed from the human neonatal rotavirus strain, RV3 (G3P[6]), identified in the stool of asymptomatic infants (10). Wild-type infection with RV3 provided protection from severe gastroenteritis in the first 3 years of life, with strong heterotypic serological responses to community rotavirus strains (11, 12). RV3 appears to be naturally attenuated and adapted to the newborn gut, replicating well despite the presence of maternal antibodies and breastfeeding (13). RV3-BB vaccine aims to take advantage of the intrinsic characteristics of this novel strain to target a birth dose vaccination strategy. RV3-BB was well tolerated and immunogenic when delivered in a neonatal or infant schedule in a phase IIa trial in New Zealand (14).

The primary objective of this study was to assess the efficacy of three doses of RV3-BB against severe rotavirus gastroenteritis to 18 months of age. Secondary objectives included assessment of efficacy, immunogenicity and safety of RV3-BB when delivered in a neonatal schedule (first dose 0-5 days of age), or an infant schedule (first dose 8-10 weeks of age), compared with placebo, efficacy to 12 months, against rotavirus gastroenteritis of any severity and all-cause severe gastroenteritis.

## Methods

### Trial design and oversight

This phase IIb, randomized, double-blind, placebo-controlled trial involving 1649 participants was conducted from January 2013 to July 2016 in primary health centers and hospitals in Central Java and Yogyakarta, Indonesia. Indonesia is a low-middle income country with an under-5 mortality rate in Yogyakarta and Central Java of 30-38 per 1000 live births and per capita gross regional product of USD $2,164-$2,326 (15, 16). Authors from Murdoch Childrens Research Institute (MCRI) and Universitas Gadjah Mada (UGM) designed the trial. The protocol was approved by the ethics committees of UGM, Royal Children's Hospital Melbourne and National Agency of Drug and Food Control, Republic of Indonesia. Use of a placebo was deemed acceptable as rotavirus vaccines are not implemented in the Indonesian National Immunization Program and cost limits private purchase (17). The study was conducted in accordance with International Council for Harmonisation Good Clinical Practice guidelines and monitored by an independent contract research organization (Quintiles Pty Ltd). The study sponsor was MCRI and Indonesian sponsor was PT Bio Farma. An independent Data Safety Monitoring Board regularly reviewed safety data. Data management was performed by Biophics, Thailand. Statistical analysis was conducted by INC Research, Australia and an independent Statistical Consultant (WR). The National Health and Medical Research Council, Bill and Melinda Gates Foundation and PT Bio Farma funded the trial but had no role in study design, data collection or interpretation, or the decision to submit for publication. The second and third authors led clinical data collection. The first author wrote the first draft of the manuscript. All authors provided review and vouch for the accuracy and completeness of the data and analysis, and for the fidelity of the trial to the protocol (available at NEJM.org).

### Participants, Randomization and Blinding

Preliminary written informed consent was obtained from pregnant women prior to cord blood collection. Final written informed consent was obtained following birth prior to confirming eligibility. Eligible infants (healthy, full term babies 0-5 days of age, birth weight of 2.5-4.0 kg) were randomized into one of three groups (neonatal vaccine group, infant vaccine group, or placebo group) in a 1:1:1 ratio according to a computer generated code (block size =6) stratified by province. Investigational product (IP) (RV3-BB or placebo) doses were drawn into syringes for dispensing by an unblinded pharmacist at the central Pharmacy in each province. Investigators, study staff, families, monitors, data managers and statisticians remained blinded throughout the study.

Participants received four 1ml oral doses of IP according to their treatment allocation, with doses administered at 0-5 days (IP dose 1), 8-10 weeks (IP dose 2), 14-16 weeks (IP dose 3) and 18-20 weeks of age (IP dose 4) (Figure 1a). IP doses 2, 3 and 4 were preceded by a 2ml dose of antacid solution (Mylanta® Original). Feeding was withheld for 30 minutes before and after each dose. IP was co-administered at the same time as vaccines in the Indonesian NIP. Participants were followed by weekly telephone contacts and monthly visits to 18 months. All participants received oral polio vaccine, except for a subset of 282 participants included in the immunogenicity analysis of RV3-BB co-administered with inactivated polio vaccine.

**Figure:**
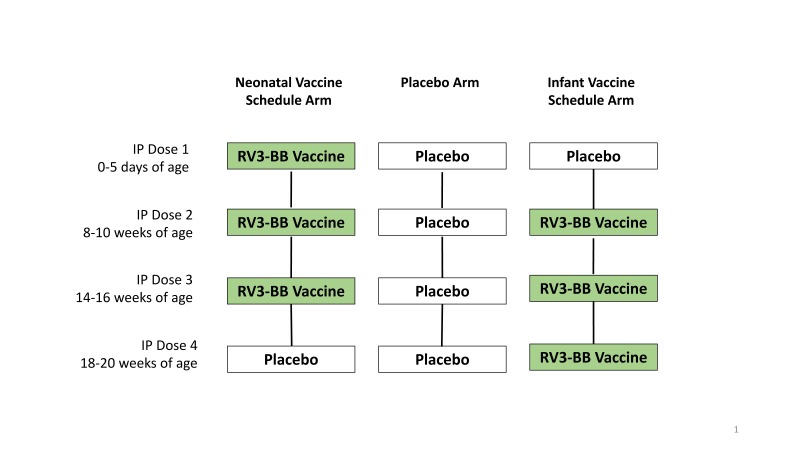


### Vaccine

RV3-BB clinical trial lots were prepared at Meridian Life Sciences (Memphis, USA) to a titre of 8.3 - 8.7 x 10^6^ FFU/mL in serum free media supplemented with 10% sucrose. Placebo contained the same media with 10% sucrose and was visually indistinguishable. Vials were stored at -70°C until thawed within 6 hours prior to administration.

### Efficacy

Severe rotavirus gastroenteritis was defined as rotavirus gastroenteritis with a modified Vesikari score of ≥ 11. Rotavirus gastroenteritis was defined as gastroenteritis with rotavirus antigen detected in the stool by enzyme linked-absorbent assay (ProSpecT Rotavirus Microplate Assay; Oxoid Ltd, UK). A modified Vesikari score was applied where intravenous, naso-gastric rehydration or 6-hours of supervised oral rehydration was scored as hospitalization, whether administered within a primary health center or hospital. Gastroenteritis of any severity, defined as three or more stools looser than normal for that child within a 24-hour period.

### Vaccine Take and Immunogenicity

Vaccine take was assessed in the first cohort recruited (n=282). Blood was collected from the cord (baseline for neonatal schedule comparison), immediately prior to IP dose 2 (baseline for infant schedule comparison), 28 days after IP dose 3 and 28 days after IP dose 4. Serum rotavirus immunoglobulin A (IgA) antibody titers and serum neutralizing antibody titers were measured using previously described methods (14, 18). RV3-BB shedding in stool was detected using a rotavirus VP6 specific reverse transcription-polymerase chain reaction assay and confirmed by sequence analysis (14). Positive vaccine take was defined as a serum immune response (≥3 fold increase in titer from baseline in anti-rotavirus IgA or serum neutralising antibodies) 28 days following IP administration, or RV3-BB shedding on days 3-7 following IP administration. Cumulative vaccine take was defined as a positive vaccine take following IP dose of 1, 2 or 3 for the neonatal vaccine group, and following IP doses 2, 3 or 4 for the infant vaccine group.

### Safety

Vital signs were assessed prior to, and in the 30 minutes after IP administration. Parents reported temperature and solicited gastrointestinal and systemic symptoms on diary cards for 7 days following each IP dose. All unsolicited adverse events (AEs) occurring up to 28 days after administration of IP doses were recorded. Serious AEs (SAEs) were defined as an AE that resulted in death, new or prolonged hospitalization or considered to be medically significant or life threatening occurring up to 28 days following the last dose of IP. Causality and severity grading of AEs were determined by the local Indonesian investigators.

### Statistical Methods

The primary efficacy analysis compared the proportion of participants with an episode of severe rotavirus gastroenteritis from two weeks after IP dose 4 to 18 months in the combined vaccine group (neonatal and infant vaccine schedules) with that observed in the placebo group in the per protocol (PP) population, using a Pearson’s Chi square test. The PP population included only participants who received all 4 doses of IP within visit windows. A secondary analysis was conducted in the intention-to-treat (ITT) population (all randomized participants), comparing events from randomisation to 18 months. Vaccine efficacy is presented as 1-risk ratio x 100 with its exact 95% confidence interval based on the Clopper-Pearson method (19).

Efficacy and vaccine take was assessed for the neonatal vaccine group (from two weeks after IP dose 3 to 12 and 18 months) and the infant vaccine group (from two weeks after IP dose 4 to 12 and 18 months). This resulted in two different presentations of data in the placebo group (denoted neonatal placebo and infant placebo). For the vaccine take analysis a participant was defined as missing only if all components of the outcome were missing. A Kaplan–Meier curve was used to estimate the cumulative hazard of a first severe rotavirus gastroenteritis episode from randomization, with group comparisons via the logrank test. All statistical tests were two-sided.

Based on local data we assumed 3% of placebo participants would experience an episode of severe rotavirus gastroenteritis during the study (20, 21) and calculated a sample size of 549 participants in each group would provide 80% power to reject the null hypothesis of no difference between the combined vaccine and placebo groups if the true efficacy was 60% (one-sided test with alpha of 0.1), allowing for 10% non-adherence. We calculated 282 participants were required to reject the null hypothesis of no difference in the proportion with a positive vaccine take (two-sided test with alpha of 0.05) assuming 25% of placebo participants would be exposed to rotavirus (14) and 50% in each vaccine group would have a positive vaccine take, allowing for 10% non-adherence.

## Results

Of the 1649 newborns randomized, 1640 received at least one dose of IP (safety population) and 1588 (96%) were followed to 18 months. The primary efficacy analysis was performed on 1513 (92%) in the PP population (Figure 1b). The demographic characteristics of the study population and age of receipt of first dose of IP were similar across all groups (Appendix: Table S1).

**Figure:**
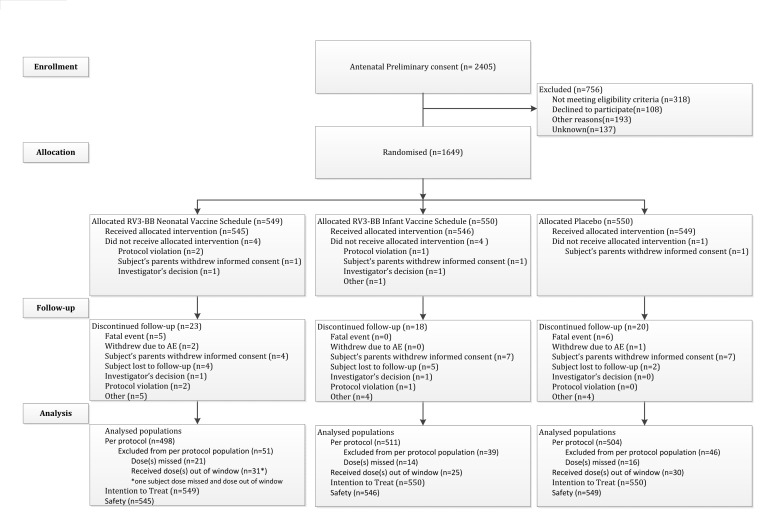


### Vaccine Efficacy

Severe rotavirus gastroenteritis occurred in 28/504 (5.6%) participants in the placebo group compared with 21/1009 (2.1%) participants in the combined vaccine group, resulting in a vaccine efficacy of 63% at 18 months in the primary (PP) analysis (95% CI, 34, 80; p<0.001), with similar results in the ITT analysis (60%; 95%CI 31, 76; <0.001) (Table 1).

**Table 1:** Efficacy of RV3-BB vaccine against severe rotavirus gastroenteritis to 18 months

	Per Protocol analysis					Intention-to-treat analysis				
	N	No. participants with an episode (%)	Efficacy*	95% CI	p value	N	No. participants with an episode (%)	Efficacy*	95% CI	p value
**Placebo**	504	28 (5.6%)				550	31 (5.6%)			
**Combined vaccine group**	1009	21 (2.1%)	63%	34, 80	<0.001	1099	25 (2.3%)	60%	31, 76	<0.001
**Neonatal vaccine group**	498	7 (1.4%)	75%	44, 91	<0.001	549	10 (1.8%)	68%	35, 86	0.001
**Infant vaccine group**	511	14 (2.7%)	51%	7, 76	0.03	550	15 (2.7%)	52%	11, 76	0.02

* When compared to respective placebo participants

When administered in the neonatal schedule, three doses of RV3-BB was associated with an efficacy of 75% against severe rotavirus gastroenteritis to 18 months (95% CI 44, 91; p<0.001) (Table 1) and 94% to 12 months (95% CI 56, 99; p=0.006) (Appendix: Table S2). Efficacy against rotavirus gastroenteritis of any severity to 18 months in the neonatal vaccine group was 63% (95% CI 37, 81; p<0.001), (Appendix: Table S2).

In the infant vaccine group, efficacy against severe rotavirus gastroenteritis to 18 months was 51% (95% CI 7, 76; p=0.03) (Table 1) and 77% to 12 months (95% CI 31, 92; p=0.008) (Appendix: Table S2). Efficacy against rotavirus gastroenteritis of any severity to 18 months when RV3-BB was administered in the infant schedule was 45% (95% CI 12, 69; p=0.01) (Appendix: Table S2).

The time from randomization to first episode of severe rotavirus gastroenteritis differed in participants receiving RV3-BB compared to placebo (Figure 2). Forty-six of 49 participants with severe rotavirus gastroenteritis had G3P[8] rotavirus detected in the stool. 

**Figure:**
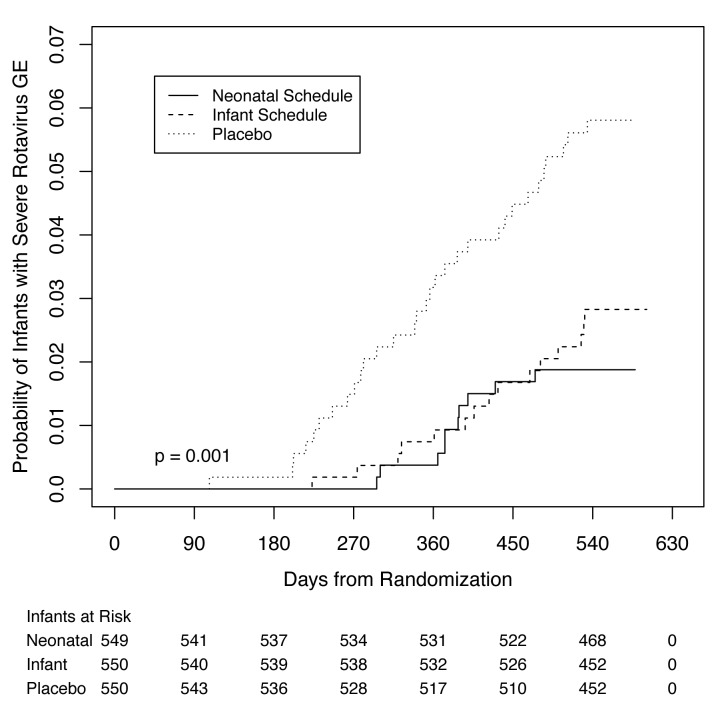


### Vaccine Take and Immunogenicity

Cumulative vaccine take following three doses of RV3-BB was detected in 78/83 (94%) of neonatal vaccine group and 83/84 (99%) of infant vaccine group (difference in proportions: neonatal vaccine group compared with neonatal placebo 0.52 [95%CI 0.39, 0.64; p<0.001]; infant vaccine group compared to infant placebo 0.52 [95%CI 0.40, 0.63; p<0.001]) (Figure 3; Appendix: Table S3). Cumulative serum immune response was observed after three doses of RV3-BB in 76% of neonatal vaccine group and 87% infant vaccine group. A serum IgA response was identified in 66% of the neonatal vaccine group and 81% of the infant vaccine group. Following two doses, cumulative vaccine take was identified in 87% of infant vaccine group compared with 28% in the infant placebo group (difference in proportions 0.59; 95% CI 0.45, 0.71: p<0.001). This comparison could not be assessed in the neonatal vaccine group as no blood was drawn at that time-point. Vaccine virus shedding was detected in 69% of the neonatal vaccine group and 75% of the infant vaccine group.

**Figure:**
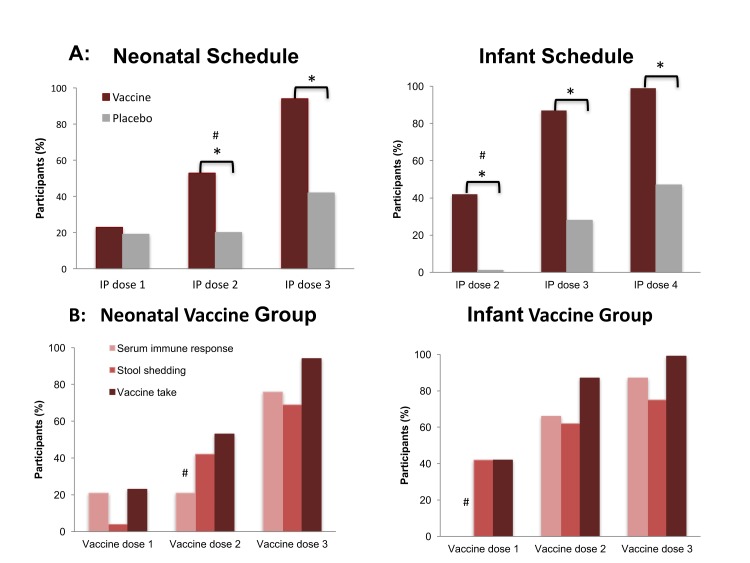


### Safety

RV3-BB was well tolerated with the incidence of SAEs (Table 2) and unsolicited and solicited AEs similar across groups (Appendix: Table S4). All 11 deaths (neonatal vaccine group n=5, placebo n=6) were assigned as unrelated to IP by the investigator and are listed in Appendix Table S5. One case of intussusception occurred 114 days after the third dose of vaccine (infant vaccine group) and was assessed as unrelated to vaccine. 

**Table 2:**
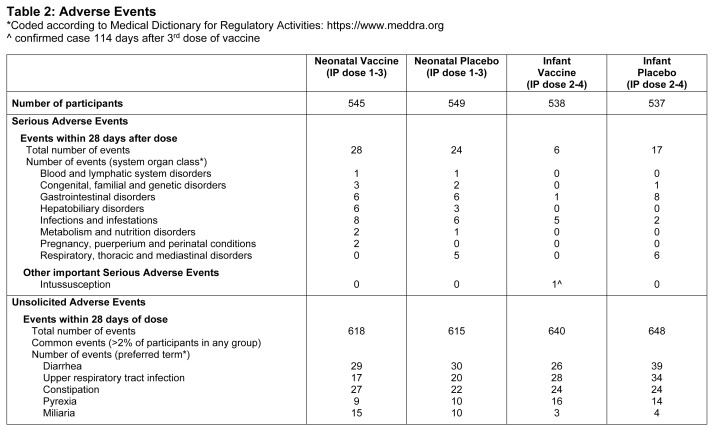
Adverse Events

## Discussion

The human neonatal vaccine RV3-BB provided protection against severe rotavirus gastroenteritis and was well tolerated. When administered in the neonatal schedule, RV3-BB had a vaccine efficacy of 94% at 12 months and 75% at 18 months, providing proof of principle for the use of RV3-BB in a birth dose vaccination schedule. These results compare very favourably with licensed vaccines studied in similar high disease burden, low- and low-middle income countries. In a two dose schedule, Rotarix (Glaxo-SmithKline) had a combined one and two year efficacy of 34% in Malawi (22). In a three dose schedule, the combined one and two year efficacy for Rotarix was 42.3% (Malawi)(22), RotaTeq (Merck) was 17.6 to 63.9% (Mali, Bangladesh, Vietnam, Ghana, and Kenya) (23, 24) and Rotavac (Bharat Biotech) was 55.1% (India) (25). Three doses of Rotasil (Serum Institute of India) had an efficacy of 66.7% at a mean follow up of 9.8 months of age in Niger (26). If the 75% protective efficacy for the neonatal schedule of RV3-BB translates into effectiveness throughout Indonesia, it has the potential to avert an estimated 5,450 deaths, 117,110 hospitalizations and >300,000 outpatient clinic visits each year due to rotavirus gastroenteritis in children under 5 years (27). 

The concept of a birth dose strategy for vaccination is not new. Birth is an established immunization time-point in many countries. A neonatal dose was investigated in the early phase of rotavirus vaccine development but not pursued due to concerns regarding inadequate immune responses and safety (28-30). The VP4 protein of human neonatal P[6] strains have specific residues at the basal surface of VP8* that may allow them to adhere to cell surface receptors in the newborn gut (31). This may provide an advantage for a birth dose schedule. The P[6] VP4 of RV3-BB may also offer an advantage in Africa and Asia where the Lewis-negative phenotype is common (32). Lewis (FUT3) and secretor (FUT2) genes appear to mediate susceptibility to rotavirus infection (32). P[8] rotaviruses only infect individuals who are Lewis-positive and secretor-positive whereas P[6] rotaviruses infect individuals irrespective of their Lewis and secretor status (33). This may explain the high proportion of disease caused by P[6] rotaviruses in Africa and the lower efficacy of vaccines with a P[8] genotype in these region (34). RV3-BB is currently the only vaccine with a P[6] VP4. 

Unlike IgG, IgA is not transferred via the placenta, and the newborn may not mount a significant serum IgA response following the birth dose of an oral vaccine, such as RV3-BB, despite evidence that the neonatal schedule is efficacious (35). Similar dissonance has been demonstrated with other vaccines administered in the newborn period (36). An equine-like G3P[8] strain was responsible for most episodes of severe gastroenteritis in this study and reflects the global emergence of this strain (37). Based on the strong heterotypic serological responses to community strains (G1,G2 dominant) provided by the parent strain RV3 (11), it is anticipated that RV3-BB will also offer protection against a range of circulating rotavirus strain but this could not be assessed in this study.

Despite the success of rotavirus vaccines remaining challenges to global implementation need to be overcome if all infants are to be protected against rotavirus disease. RV3-BB was efficacious, immunogenic and well-tolerated when administered in a neonatal or infant schedule. In particular, the high protective efficacy in the neonatal schedule suggests that RV3-BB could make a significant contribution to the global prevention of rotavirus disease. 

## Supplementary Appendix

Supplementary Material
