# Supplementary material for: Human neonatal rotavirus vaccine (RV3-BB) targets rotavirus from birth
Source: N Engl J Med. Author manuscript; Available in PMC 2018 Feb 22. (PMC5774175; doi:10.1056/NEJMoa1706804)
Supplement: Supplementary Material [file NEJMoa1706804Bines_SuppApp.pdf]

SUPPLEMENTARY APPENDIX

Human neonatal rotavirus vaccine (RV3-BB) targets rotavirus from birth

Julie E. Bines, M.D. [1,2,3]
Jarir At Thobari, Ph.D.[4]
Cahya Dewi Satria, M.D.[4]
*Amanda Handley, M.P.H. [1,5]
*Emma Watts, B. Sci. (Hons) [1]
Daniel Cowley, Ph.D. [1,2]
Hera Nirwati, M.D., Ph. D.[4]
James Ackland, B.Sci. [6]
Jane Standish, M.B.B.S. [1,3]
Frances Justice, B.Sci. [1]
Gabrielle Byars, MBioMedSci[1]
Katherine J. Lee, Ph.D.[1,2]
Graeme L. Barnes, M.D. [1,2]
Novilia S. Bachtiar, dr M.Kes.[7]
Ajeng Viska Icanervilia M.D.[4]
Karen Boniface, B.Sci (Hons)[1]
Nada Bogdanovic-Sakran, B.Sci [1]
Daniel Pavlic, B.Sci (Hons) [1]
Ruth F. Bishop, A.O., D. Sc. [1,2]
Carl D. Kirkwood, Ph.D. [1,2,8]
Jim P. Buttery, M.D. [1,2,9]
Yati Soenarto, M.D., D.Sc [4]

Joint authors

From the:
[1]RV3 Rotavirus Vaccine Program, Murdoch Childrens Research Institute, Parkville, Victoria, Australia (J.E.B., A.H., D.C., E.W., C.D.K., J.S., K.J.L, F.J., G.B., K.B., N.B., D.P., G.L.B., R.F.B., J.P.B.)

[2]Department of Paediatrics, The University of Melbourne (J.E.B., D.C., C.D.K., K.J.L., G.L.B., R.F.B., J.P.B.)
[3]Department of Gastroenterology and Clinical Nutrition, Royal Children's Hospital (J.E.B., J.S.)
[4]Pediatric Research Office, Department of Paediatrics (C.D.S, A.V.I., Y.S.), Department of Pharmacology and Therapy (J.A.T.) and Department of Microbiology (H.N.W.); Faculty of Medicine, Universitas Gadjah Mada, Yogyakarta
[5]Medicines Development for Global Health, Melbourne, Australia (A.H)
[6]Global BioSolutions, Melbourne, Australia (J.A.)
[7]PT Bio Farma, Bandung, Indonesia (N.S.B.)
[8]Bill and Melinda Gates Foundation, Seattle, U.S.A. (C.D.K.)
[9]Departments of Paediatrics and Epidemiology & Preventive Medicine, Monash University; Department of Infection & Immunity, Monash Children's Hospital, Clayton, Victoria, Australia (J.P.B.)

Corresponding author:
Professor Julie Bines
The University of Melbourne
Level 2, Royal Children's Hospital, 50 Flemington Rd,
Parkville, 3052 Victoria, Australia
Email: jebines@unimelb.edu.au

Table of Contents:

Table S1: Demographic characteristics of the Study Population Page 4

Table S1A: PP population

Table S1B: ITT population

Table S1C: Safety population

Table S2: Additional secondary analyses of RV3-BB Vaccine Efficacy Page 7

Table S3: Vaccine take and Components of Vaccine Take Page 9

Table S4A Neonatal Schedule

Table S4B Infant Schedule

Table S4: Adverse Events in the Safety Population Page 11

Table S5: Fatal Events Page 13

TABLE S1A: DEMOGRAPHIC CHARACTERISTICS OF STUDY POPULATION (PP POPULATION)

	Neonatal Vaccine Schedule (N=498)	Infant Vaccine Schedule (N=511)	Placebo (N=504)
Age at randomization (days)			
Mean (SD)	3.4 (1.3)	3.5 (1.3)	3.5 (1.3)
Sex			
Male	270 (54.2%)	265 (51.9%)	256 (50.8%)
Ethnicity			
Javanese	497 (99.8%)	509 (99.6%)	502 (99.6%)
Sundanese	0 (0.0%)	0 (0.0%)	1 (0.2%)
Chinese	0 (0.0%)	1 (0.2%)	0 (0.0%)
Malaya	0 (0.0%)	0 (0.0%)	0 (0.0%)
Other	1 (0.2%)	1 (0.2%)	1 (0.2%)
Gestational Age (weeks)			
Mean (SD)	39.55 (1.10)	39.53 (1.08)	39.54 (1.12)
Birth Weight (g)			
Mean (SD)	3117.57 (347.65)	3136.01 (333.55)	3106.25 (338.35)
Height/Length (cm)			
Mean (SD)	48.70 (1.65)	48.65 (1.67)	48.62 (1.68)

Table S1B: DEMOGRAPHIC CHARACTERISTICS OF STUDY POPULATION (ITT POPULATION)

	Neonatal Vaccine Schedule (N=549)	Infant Vaccine Schedule (N=550)	Placebo (N=550)
Age at randomization (days)			
Mean (SD)	3.4 (1.3)	3.5 (1.3)	3.5 (1.3)
Sex			
Male	297 (54.1%)	288 (52.4%)	279 (50.7%)
Ethnicity			
Javanese	548 (99.8%)	548 (99.6%)	548 (99.6%)
Sundanese	0 (0.0%)	0 (0.0%)	1 (0.2%)
Chinese	0 (0.0%)	1 (0.2%)	0 (0.0%)
Malaya	0 (0.0%)	0 (0.0%)	0 (0.0%)
Gestational Age (weeks)			
Mean (SD)	39.55 (1.11)	39.51 (1.10)	39.52 (1.12)
Weight (g)			
Mean (SD)	3118.16 (348.41)	3127.22 (331.47)	3103.69 (336.98)
Height/Length (cm)			
Mean (SD)	48.67 (1.65)	48.64 (1.66)	48.63 (1.67)

Table S1C: DEMOGRAPHIC CHARACTERISTICS OF STUDY POPULATION (SAFETY POPULATION)

	Neonatal Vaccine Schedule (N=545)	Infant Vaccine Schedule (N=546)	Placebo (N=549)
Age at randomization (days)			
Mean (SD)	3.4 (1.3)	3.5 (1.3)	3.5 (1.3)
Sex			
Male	296 (54.3%)	285 (52.2%)	278 (50.6%)
Ethnicity			
Javanese	544 (99.8%)	544 (99.6%)	547 (99.6%)
Sundanese	0 (0.0%)	0 (0.0%)	1 (0.2%)
Chinese	0 (0.0%)	1 (0.2%)	0 (0.0%)
Malaya	0 (0.0%)	0 (0.0%)	0 (0.0%)
Other	1 (0.2%)	1 (0.2%)	1 (0.2%)
Gestational Age (weeks)			
Mean (SD)	39.54 (1.11)	39.51 (1.10)	39.51 (1.12)
Birth Weight (g)			
Mean (SD)	3115.36 (345.65)	3129.21 (330.71)	3102.97 (336.86)
Height/Length (cm)			
Mean (SD)	48.66 (1.64)	48.63 (1.66)	48.63 (1.68)

TABLE S2: Additional secondary analyses of RV3-BB Vaccine Efficacy

EFFICACY OF RV3-BB VACCINE AGAINST SEVERE ROTAVIRUS GASTROENTERITIS TO 12 MONTHS OF AGE (PER PROTOCOL ANALYSIS)					
	**N**	**No. participants with an episode (%)**	**Efficacy***	**95% CI**	**p value**
**Placebo**	504	17 (3.4%)			
**Combined Vaccine Group**	1009	5 (0.5%)	85%	60, 95	<0.001
**Neonatal Vaccine Group**	498	1 (0.2%)	94%	55, 99	0.006
**Infant Vaccine Group**	511	4 (0.8%)	77%	32, 92	0.008
**EFFICACY OF RV3-BB VACCINE AGAINST ROTAVIRUS GASTROENTERITIS TO 18 MONTHS OF AGE (PER PROTOCOL ANALYSIS)**					
	**N**	**No. participants with an episode (%)**	**Efficacy***	**95% CI**	**p value**
**Placebo**	504	47 (9.3%)			
**Combined Vaccine Group**	1009	43 (4.3%)	54%	31,70	<0.001
**Neonatal Vaccine Group**	498	17 (3.4%)	63%	37,81	<0.001
**Infant Vaccine Group**	511	26 (5.1%)	45%	12,69	0.01

EFFICACY OF RV3-BB VACCINE AGAINST ALL-CAUSE SEVERE GASTROENTERITIS TO 18 MONTHS OF AGE (PER PROTOCOL ANALYSIS)					
	**N**	**No. participants with an episode (%)**	**Efficacy***	**95% CI**	**p value**
**Placebo**	504	55 (10.9%)			
**Combined Vaccine Group**	1009	82 (8.1%)	26%	-5,46	0.09
**Neonatal Vaccine Group**	498	42 (8.4%)	27%	-7,52	0.11
**Infant Vaccine Group**	511	40 (7.8%)	28%	-6,53	0.11

* when compared to respective placebo participants

Table S3: Cumulative vaccine take in Immunogenicity subset (per-protocol population)

A) Neonatal vaccine schedule comparison

	Neonatal Vaccine Group	Neonatal Placebo Group	Difference in proportions (95% CI)	p value
Dose 1: 0-5 days of age IP dose				
Serum Response	17/82 (21%)	14/77 (18%)		
Serum IgA	17/82 (21%)	14/77 (18%)		
SNA	0/82 (0%)	0/77(0%)		
Stool excretion	3/82 (4%)	2/78 (3%)		
Vaccine Take	19/83 (23%)	15/79 (19%)		>0.01
Dose 2: 8-10 weeks of age IP dose^				
Cumulative Stool excretion	35/83 (42%)	3/79 (4%)		
Cumulative Vaccine Take	44/83 (53%)*	16/79 (20%)*		<0.001
Dose 3: 14-16 weeks of age IP dose				
Cumulative Serum Response	63/83 (76%)	32/78 (41%)	0.35 (0.18, 0.49)	<0.001
Cumulative Serum IgA	55/83 (66%)	31/78 (40%)	0.27 (0.10, 0.41)	<0.001
Cumulative SNA	20/83 (24%)	4/78 (5%)	0.19 (0.07,0.30)	<0.001
Cumulative Stool excretion	57/83 (69%)	4/79 (5%)	0.64 (0.51, 0.74)	<0.001
Cumulative Vaccine Take	78/83 (94%)	33/79 (42%)	0.52 (0.39, 0.64)	<0.001

*No assessment of serum immune response conducted at this time-point

B) Infant vaccine schedule comparison

	Infant Vaccine Schedule	Infant Placebo Group	Difference in proportions (95% CI)	p value
**Dose 1: 8-10 weeks of age^**				
Stool excretion	33/78 (42%)	1/77 (1%)		
Vaccine Take	33/78 (42%)*	1/77 (1%)*		<0.001
**Dose 2: 14-16 weeks of age**				
Serum Response	55/84 (66%)	22/78 (28%)		
Serum IgA	52/84 (62%)	19/78 (24%)		
SNA	11/84 (13%)	4/77 (5%)		
Cumulative stool excretion	52/84 (62%)	2/78 (3%)		
Cumulative Vaccine Take	73/84 (87%)	22/79 (28%)	0.59 (0.45, 0.71)	<0.001
**Dose 3: 20-24 weeks of age**				
Cumulative Serum Response	73/84 (87%)	37/78 (47%)	0.39 (0.24, 0.53)	<0.001
Cumulative Serum IgA	68/84 (81%)	33/78 (42%)	0.39 (0.23, 0.52)	<0.001
Cumulative SNA	32/84 (38%)	6/78 (8%)	0.30 (0.17, 0.43)	<0.001
Cumulative stool excretion	63/84 (75%)	2/78 (3%)	0.72 (0.61, 0.82)	<0.001
Cumulative Vaccine Take	83/84 (99%)	37/79 (47%)	0.52 (0.40, 0.63)	<0.001

*No assessment of serum immune response conducted at this time-point

Table S4: Adverse Events reported in Safety Population

Safety population	Neonatal Vaccine schedule (IP dose 1-3) N=545	Neonatal Placebo schedule (IP dose 1-3) N=549	Infant Vaccine Schedule (IP dose 2-4) N=538	Infant Placebo Schedule (IP dose 2-4) N=537
**Unsolicited Adverse Events (0 to 28 days post dose)**				
Subjects with at least one unsolicited AE	311	330	323	329
Subjects with at least one ≥Grade 3/severe unsolicited AE assessed as possibly, probably or definitely related to IP	2	1	0	0
Subjects with at least one unsolicited AE assessed as possibly, probably or definitely related to IP	107	109	98	111
Total number of unsolicited AEs	618	615	640	648
Total number of ≥Grade 3/severe unsolicited AEs assessed as possibly, probably or definitely related to IP	4	1	0	0
Total number of unsolicited AEs assessed as possibly, probably or definitely related to IP	183	182	169	187
**Solicited Adverse Events (0-7 days post dose)**				
Subjects with at least one solicited AE	400	388	395	387

Subjects with at least one ≥Grade 3/severe solicited AE assessed as possibly, probably or definitely related to IP	1	0	0	0
Subjects with at least one solicited AE assessed as possibly, probably or definitely related to IP	272	280	285	282
Total number of solicited AEs	1262	1239	1475	1470
Total number of ≥Grade 3/severe solicited AEs assessed as possibly, probably or definitely related to IP	3	0	0	0
Total number of solicited AEs assessed as possibly, probably or definitely related to IP	816	829	983	1010

Table S5: Fatal Events

**Fatal Serious Adverse Events (Preferred term)**	**Neonatal Schedule**	**Infant Schedule**	**Placebo**
**<28 days post any IP dose**			
Persistent fetal circulation	0	0	1
Volvulus	0	0	1
Encephalitis	1	0	0
Neonatal aspiration	0	0	1
**>28 days post IP dose**			
Ventricular Septal Defect	1	0	0
Septic shock	2	0	1
Brain mass	1	0	0
Fallot's Tetralogy	0	0	1
Dengue fever	0	0	1
**Total**	**5**	**0**	**6**
